# Microbubbles Ultrasonic Cavitation Regulates Tumor Interstitial Fluid Pressure and Enhances Sonodynamic Therapy

**DOI:** 10.3389/fonc.2022.852454

**Published:** 2022-04-26

**Authors:** Fen Xi, Yuyi Feng, Qiaoli Chen, Liping Chen, Jianhua Liu

**Affiliations:** ^1^ The First Affiliated Hospital, Jinan University, Guangzhou, China; ^2^ Department of Ultrasound Medical, Guangzhou First People’s Hospital, Guangzhou Medical University, Guangzhou, China; ^3^ The Second Affiliated Hospital, School of Medicine, South China University of Technology, Guangzhou, China

**Keywords:** ultrasound, microbubbles, cavitation, tumor interstitial fluid pressure, SDT

## Abstract

Sonodynamic therapy (SDT) is a promising treatment method for solid tumors. However, the high interstitial fluid pressure (IFP) in tumor tissues limits the accumulation of sonosensitizers. In the present study, microbubbles ultrasonic cavitation was used to regulate the tumor’s IFP and evaluate SDT effects. Rabbit VX2 tumor tissues were treated with microbubbles ultrasonic cavitation. The IFP of different tumor parts before and after cavitation was measured by the WIN method. The accumulation of the sonosensitizers hematoporphyrin monomethyl ether (HMME) in tumor tissues was observed using an ultramicro spectrophotometer and laser confocal microscope. Then, tumor-bearing rabbits were treated with SDT once a week for eight weeks and the therapeutic effect was evaluated. After microbubbles ultrasonic cavitation treatment, the tumor’s IFP decreased and the HMME concentration increased. We concluded that microbubbles ultrasonic cavitation can increase HMME accumulation in rabbit VX2 tumors and increase SDT therapeutic effects.

## Introduction

Tumors cause important diseases threatening to human health, and their treatment methods are still developing, including surgery, chemotherapy alone or in combination, radiotherapy, interventional therapy, microwave ablation, immunotherapy etc. Among them Sonodynamic therapy (SDT) (1) is a new tumor treatment strategy and have advantages such as safety, being non-invasive, and having good targeting and great clinical application prospects. SDT mainly refers to the irradiation of the tumor site with ultrasound of specific frequencies and intensities for a certain time to activate ultrasound-sensitive drugs enriched in tumor tissues. This can significantly enhance drugs’ cytotoxicity in the targeted area and specifically kill tumor cells. Compared with photodynamic therapy (PDT), SDT has a deeper tissue penetration. Some studies (2) have shown that after A549 tumor-bearing mice were irradiated with pulsed focused ultrasound (5 W/cm2), the dense and hard extracellular matrix became loose, collagen fibers were destroyed, and the targeting and penetration of nanoparticles were significantly enhanced.

Hematoporphyrin monomethyl ether (HMME) (3–5) is a second-generation porphyrin sonosensitizer and has advantages such as single composition, stable performance, high tumor selectivity, and low phototoxicity to normal tissues. Its concentration in tumor has an important effect on the therapeutic effect of SDT. In many cases, the delivery of sonosensitizers or specific targeted drugs to tumors uses the vascular system, but many solid tumors have abnormal vascular structures, lymphatic dysfunctions, and extracellular matrix components imbalances, increasing the interstitial fluid pressure (IFP) (6, 7) which is one of the important reasons for the low accumulation of acoustic sensitizers in tumor tissues. In normal tissues (8), the IFP is about -1~3 mmHg (7) but animal and human tumors present higher IFPs (9–11) about 30 mmHg in breast cancer, and even more in cervical cancer, metastatic melanoma, colon cancer liver metastases, head, and neck tumors. At the same time, studies (12) have shown that a high IFP is related to reduced radiotherapy sensitivity and insufficient uptake of chemotherapy drugs. Therefore, Reducing IFP to increase the permeability of HMME in tumor tissue and increase the effective concentration of HMME can be one of the breakthroughs to enhance SDT.

**Figure 1:**
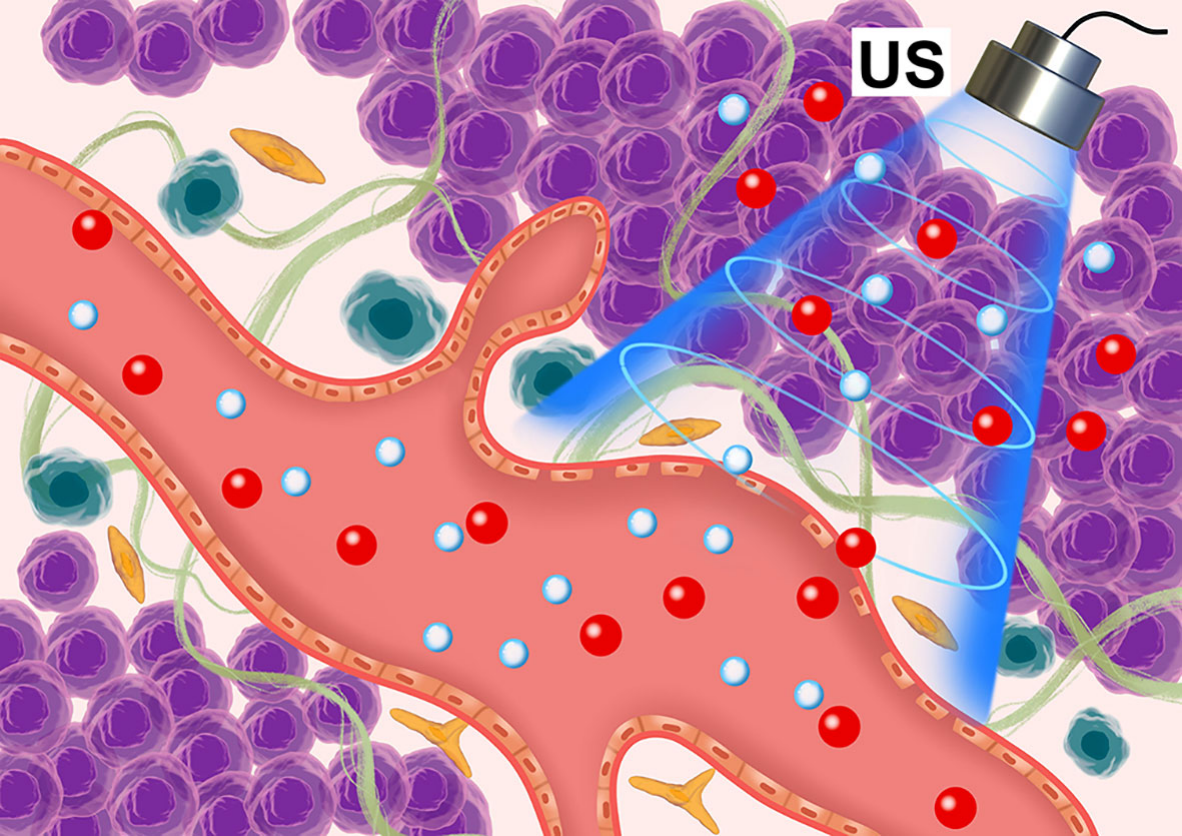
Schematic diagram of the microbubble ultrasonic cavitation pretreatment with the ultrasound-sensitizer HMME.

Microbubbles are spheres (diameter 1-10 μm) composed of polymers, proteins, or thin lipid shells filled with inert gas. They have cavitation effects under ultrasonic irradiation, producing shock waves and microjets, which can destroy tumor microvascular structures, damage endothelial cells, and even cause cell lysis (13, 14). Therefore, through ultrasonic cavitation, blood vessels can be destroyed and embolized, tumor cell apoptosis and necrosis induced, tumor cell density reduced, tissue space expanded, and the IFP reduced. In a previous study (15, 16) we established a VX2 transplanted tumor model in the superficial muscle layer of a rabbit left hind limb and selected low-frequency unfocused ultrasounds combined with microbubbles to irradiate the tumor (ultrasound parameters: center frequency 1 MHz; ultrasound pressure 1, 3, and 5 MPa; pulse repetition frequency 10 Hz; duty cycle 0.2%; pulse emission/gap time 9 s/3 s). Results showed that medium-high ultrasound pressure (3 and 5 MPa) and low-frequency unfocused ultrasound irradiation for 5 min decreased the IFP. Lower ultrasound pressure (1 MPa) prolonging the irradiation time for 10 min also led to tumor IFP decrease. Hence, in the present study, we used microbubbles ultrasonic cavitation biological effects to regulate tumors IFP and explore the best therapeutic ultrasound parameters to improve the permeability of sonosensitizers in tumors, and finally, increase SDT effects and analyze its possible mechanisms **(** **Figure 1** **)**.

## Materials And Methods

### Reagents and Animal Models

The HMME was purchased from Shanghai Dibo Biotechnology Co., Ltd., China, and was stored in a refrigerator protected from light at 4°C. Microbubbles for injection (SonoVue) were purchased from Bracco Suisse SA Italy. The main components of SonoVue are sulfur hexafluoride gas and phospholipid. The average diameter is 2.5 μm and the diameter of 90% microbubbles is less than 6 μm with very low solubility in blood, and can be exhaled through microcirculation.

Healthy adult female New Zealand White rabbits (2.0-2.5 kg) were purchased from the experimental animal center of Guangdong Province. They were adapted in a suitable feeding environment for 7 d. After weighing, they were anesthetized by compound anesthesia. The anesthesia was intramuscularly injected and consisted of 0.15 mL/kg Sumianxin II and 20 mg/kg 2% Pentobarbital sodium. After the corneal reflex disappeared, rabbits were fixed in the lateral position on the experimental table and the skin of the left hind limb was prepared. The rabbit’s VX2 tumor tissue block was cut to 1 mm^3^ and placed in normal saline to form a suspension. Then, a 1 mL syringe was used to connect the G needle, 1~2 tissue blocks were sucked and injected into the superficial muscle layer of the left hind limb of the rabbit (depth: 2.5 ± 0.5 mm from the body surface). The tumor size of rabbits was observed by ultrasonic diagnostic instrument (GE LOGIQ E9, probe: ML6-15) every day and grew to L 10 ± 0.7 mm and W 5 ± 0.8 mm in about 10 d. All animal experiments were carried out following the guidelines of the National Institute of Health. The care and use of experimental animals were approved by the animal ethics committee of the South China University of Technology.

### Contrast-Enhanced Ultrasound Examination

**Figure 2:**
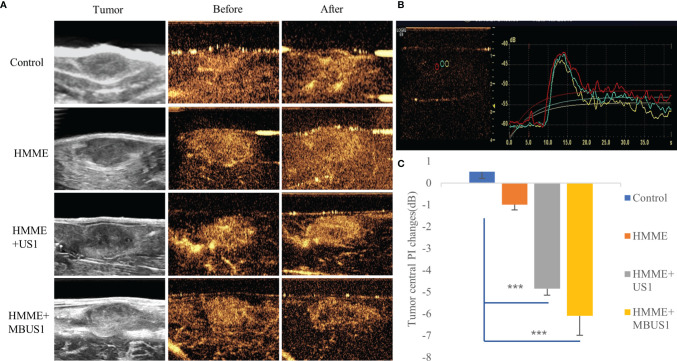
**(A)** Peak intensity contrast images of each group before and after ultrasound irradiation. **(B)** The ultrasound diagnostic instrument quantitatively analyzed the contrast intensity at the center (yellow), edge 1/4 (green), and edge 1/8 (red). **(C)** After ultrasound irradiation, the PI changes in the tumor’s central part in each group (***p < 0.001).

All tumor-bearing rabbits received contrast-enhanced ultrasound before and after microbubbles ultrasonic cavitation treatment. The Color Doppler ultrasound diagnostic instrument (GE LOGIQ E9, probe: ML6-15) was used in the contrast-enhanced ultrasound mode. SonoVue microbubbles were diluted with 0.2 mL normal saline, then injected through rabbit ear marginal vein by mass injection. Next, the normal saline flushing tube was connected, and the angiography started. The dynamic image was recorded and stored for 1 min, and the angiography peak intensity (PI) was recorded and analyzed **(** **Figure 2B** **)**.

### Microbubbles Ultrasonic Cavitation Therapy

Twenty tumor-bearing rabbits without defects related to the above contrast medium were divided into four groups (five rabbits in each group): HMME + MBUS1, HMME + US1, HMME, and blank control. Each group was treated as follows: in the HMME + MBUS1 group, each rabbit was intravenously injected with 5.0 mg/kg HMME at the ear margin 1 h later, ultrasonic emission frequency of 2.5 MPa, pulse repetition frequency of 10 Hz, a duty cycle of 0.2%, pulse emission/gap time of 9 s/3 s (The choice of this parameter is based on prior research that we are currently publishing), and irradiation time of 5 min(Shenzhen Wilde Medical Electronics Co., Ltd., models dct-700 and kht-017; effective diameter 20 mm). The probe irradiated the tumor and the SonoVue microbubbles diluted (5 mL with sterile normal saline) were slowly injected (0.5 mL/kg); in the HMME + US1 group, after each tumor-bearing rabbit was injected with the same HMME dose for 1 h, the ultrasound treatment probe was irradiated and the same volume of normal saline was slowly injected; in the HMME group, after each tumor-bearing rabbit was injected with the same HMME dose for 1 h, the ultrasound was sham irradiated for 5 min; in the blank control group, the tumor-bearing rabbits were injected with the same volume of normal saline for 1 h, then the ultrasound was sham irradiated for 5 min. The tumor IFP was measured by the WIN method before and after ultrasonic treatments.

### Tumor IFP Measurement

**Figure 3:**
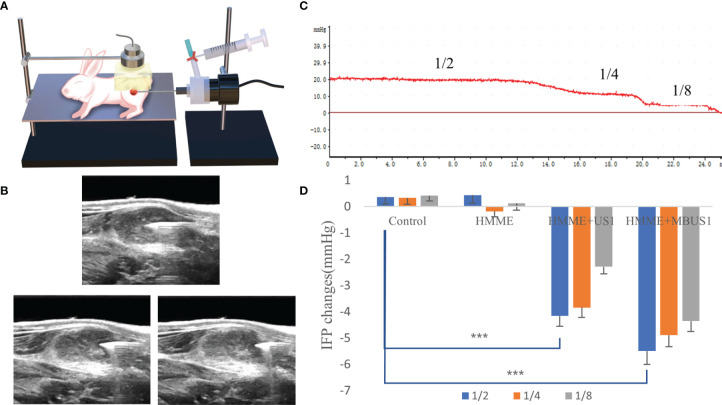
**(A)** The VX2 tumor pattern was measured by ultrasound irradiation and the WIN method. **(B)** Schematic diagram of needle core position. IFP of 1/2, 1/4, and 1/8 tumors were respectively measured. **(C)** Schematic diagram of IFP measurements of a single tumor. The values corresponding to different steps are the IFP values at that point. **(D)** Changes of IFP values at different tumor locations after ultrasound irradiation (***p < 0.001).

The WIN method (17) was used to measure the tumor’s IFP (**Figure 3A**). The central and peripheral regions of the tumor were distinguished by taking 1/4 of the tumor diameter at the boundary point. At different time nodes, immediately before and after each treatment, the WIN method was used to measure the three tumor regions (the central 1/2, the marginal 1/4, and the peripheral 1/8) **(** **Figure 3B** **)**. All measurements were repeated three times and averaged as the results. First, the instrument connected the puncture needle with a side hole to the biological signal acquisition and analysis system, filled the hydraulic measurement system with heparin sodium saline sealing solution, was calibrated and blanked before measurements, and was horizontally placed on the horizontal plane at the same height as the tumor. Then, the puncture needle entered the center of the tumor under ultrasound guidance and marked the curve after the pressure curve was stably displayed for 1 min. At the beginning of the measurement, the pressure was recorded for 10 s, and the curve marked the end. The average reading of the pressure curve within 10 s was used as the initial IFP value of the tumor. After treatments, the puncture needle was slowly withdrawn and the IFP was measured in the peripheral area of the tumor and the normal tissue around it by the same method. Measurement results were determined by the bl-420s biological function experimental system (developed by Chengdu taimeng Software Co, Ltd.; model BL-420s. Pressure sensor model FT-100s).

### HMME Content and Distribution in Tumors

After the above measurements, all tumor-bearing rabbits were euthanized and the tumor was removed. The tumor tissues were divided into four parts and fixed with paraformaldehyde then stored away from light. The tumors 1/4~1/8 near the edge were accurately weighed (1 g), 1 mL of normal saline homogenate was added and grinded, shaken for 15 min, and centrifuged (3000 rpm for 10 min). The supernatant was recovered and the fluorescence intensity was measured with an ultra microspectrophotometer (American DeNovix model DS ll +). The standard concentration curve consisted of different HMME concentrations.

A portion of tumor tissues shielded from light were sliced, then DAPI (excitation wavelength 340 nm, emission wavelength 488 nm) was used to stain the tumor nucleus, and the HMME distribution (excitation wavelength 395nm, emission wavelength 611nm) in the tumor tissue was observed by confocal laser microscopy (Japan Nikon Ti-E-A1).

### Pathological Analyses

Half of each tumor was sliced for staining analysis, HE staining was used to observe tumor vascular permeability and surrounding changes. The Masson and Gordon sweets reticular fiber stainings were used to observe the changes of collagen fibers and reticular microscopic content in tumor tissues after ultrasonic treatments. Pathological sections were scanned by a pathological scanner (Chinese SDPTOP, model HS6) and observed by image scope software.

### SDT

Twenty tumor-bearing rabbits were divided into four groups (five rabbits in each group): HMME + MBUS1 + SDT, HMME + SDT, HMME + MBUS1, and blank control. The rabbits in each group were treated once a week according to different treatment methods. The HMME dose, MBUS parameters, and instruments were the same as before, and the SDT instrument (Shenzhen Wilde Medical Electronics Co., Ltd., model wed-100) had an effective probe diameter of 20 mm, pulse waveform, ultrasonic frequency of 1 MHz, an ultrasonic intensity of 3 W/cm^2^, a duty cycle of 60%, and the treatment time was 15 min. The length, width, and thickness of the tumor were measured by an ultrasonic diagnostic instrument once two days, according to the formula: v = L×W×H×π/6, where: length (L), width (W), and height (H) were used to calculate the tumor volume (V) and draw the tumor growth curve. All measurements were repeated three times and averaged as the results. After eight weeks of treatment in February, the tumor-bearing rabbits in different groups were euthanized and weighed before the tumor removed (**Figure 6A**). The lung and liver were also removed. They were fixed with paraformaldehyde, embedded in paraffin, cut, and stained. The histopathological changes and metastasis were observed under an optical microscope.

### Statistics

The SPSS 22.0 software was used for statistical analyses. The measured values are expressed as means ± standard deviations (SDs). Pairwise comparisons were determined using the t-test. The differences between groups were determined by the one-way analysis of variance (ANOVA) test, and the homogeneity of variance test was performed before analysis. *p*<0.05 was considered to be statistically significant.

## Results

### Effect of Microbubbles Ultrasonic Cavitation on Tumor Blood Perfusion Evaluated by Contrast-Enhanced Ultrasound

Before treatments, the contrast-enhanced ultrasound showed that, in each group, the microbubbles were rapidly and evenly filled in the tumor and reached the peak value in about 12 s. No filling defect was detected. After the 2.5 MPa ultrasound irradiation, we quantitatively analyzed the peak intensity at the tumor’s center 1/2 and the edge 1/4 and 1/8 **(** **Figure 2B** **)**. The square difference homogeneity test was performed in each group and all presented a *p >* 0.05. Moreover, the one-way ANOVA results were significant. In the central part of the tumor, the PI values of the HMME, HMME + MBUS1, and HMME + MBUS1 groups significantly decreased in varying degrees different (*F* = 18.384*, p* = 0.000). In the HMME + MBUS1 group, a decrease of 6.01 dB was detected, with a 10.01% degree before angiography. However, we detected a little filling defect in the central part of the tumor in the HMME + MBUS1 and HMME + US1 groups under naked-eye observations **(** **Figure 2A** **)**. Additionally, the HMME + MBUS1 defective area was larger compared to the HMME + US1 group. After treatments, the contrast image in the HMME group could not be distinguished by the naked eye, but the PI value in the central part decreased slightly, while the qualitative and quantitative scores in the blank control group did not significantly change (**Figure 2C**). No significant changes were detected At the tumor’s edge 1/4 (*F* = 2.717*, p* = 0.079) and 1/8 (*F* = 2.849*, p* = 2.070) in tumor PI values before and after ultrasound irradiation. Finally, the contrast medium was well filled in the above parts of the image, and no significant differences before and after irradiation were detected for the tumor’s center 1/2 (*p* = 0.236), and edge 1/4 (*p* = 0.140), and 1/8 (*p* = 0.071).

### Tumor IFP Changes After Microbubbles Ultrasonic Cavitation

Before ultrasonic cavitation, when the puncture needle was stably placed in the center of the tumor and moved outward to 1/2 and 1/8 (**Figure 3**), the tumor IFP waveform curve in each group showed a positive and stepped form (**Figure 3C**). Before treatment, the average tumor IFP value at the center 1/2, and the edge 1/4 and 1/8 of the 20 tumor-bearing rabbits were (means ± SDs) 16.76 ± 2.77, 11.42 ± 2.25, and 4.65 ± 1.94 mmHg, respectively. Also, when the ultrasound needle moved out from the edge of the tumor to the muscle tissue, the curve decreased to zero and negative values. After cavitation irradiation treatment, the puncture needle punctured the same part of the tumor. Results showed that in the three different tumor parts, the tumor IFP decreased in different degrees in HMME + US1 and HMME + MBUS1 groups. The decrease was more clear in the HMME + MBUS1 group, followed by HMME + US1. Meanwhile, in blank control and HMME groups (sham irradiation groups), the IFP in each part of the tumor did not significantly change. Overall, there were significant differences among groups (all *p* = 0.000). After irradiation, The ΔIFP values of HMME + US1 and HMME + MBUS1 groups were the highest in the center (-4.15 ± 1.81 and -5.50 ± 2.47 mmHg, respectively), 25 and 32.8% lower than those before treatment (**Figure 3D**). The IFP values of the two edge positions also decreased, and the decline degree of the HMME + MBUS1 was 42.8 (1/4) and 93.5% (1/8). The IFP at the tumor’s edge 1/4 and 1/8 of the HMME + US1 group decreased by 33.6 and 83%, respectively, compared with the values before ultrasonic irradiation. Although the IFP value at the edge was relatively small, the decline rate was related to the low IFP before ultrasonic irradiation (p < 0.05).

### HMME Content in Tumor Tissues After Ultrasound Irradiation

**Figure 4:**
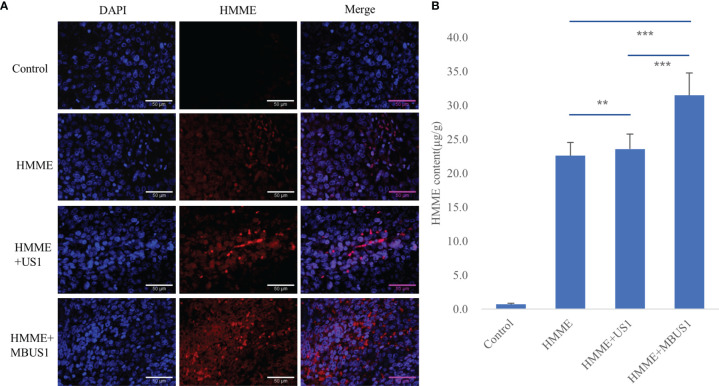
**(A)** DAPI staining images of tumors under laser confocal microscope. Blue represents the nucleus and red the HMME. **(B)** The HMME content in tumor tissues was quantitatively analyzed by an ultramicro photometer (**p < 0.01,***p <0.001).

We used two methods to qualitatively and quantitatively analyze the HMME content in tumor tissues. The laser confocal image under DAPI staining showed that a little red fluorescence could be seen in tumor tissues after intravenous HMME injection, which was mainly concentrated outside tumor cells (**Figure 4A**). The intracellular and extracellular red fluorescence increased in the HMME + US1 group. Also, the red fluorescence in tumor tissues increased in the HMME + MBUS1 group due to the addition of microbubbles The HMME content in the tumor tissue of each treatment group significantly increased over time (*F* = 32.221, *p* = 0.000). The HMME concentration in tumor tissues of the HMME + MBUS1 group was the highest, reaching 31.5 μg/g, comprehending a 31% increase compared to the HMME group. In the HMME + US1 group (without microbubbles), the HMME content in the tumor tissue only increased by 4% (**Figure 4** **B**).

### Pathological Changes of Tumor Sections

**Figure 5:**
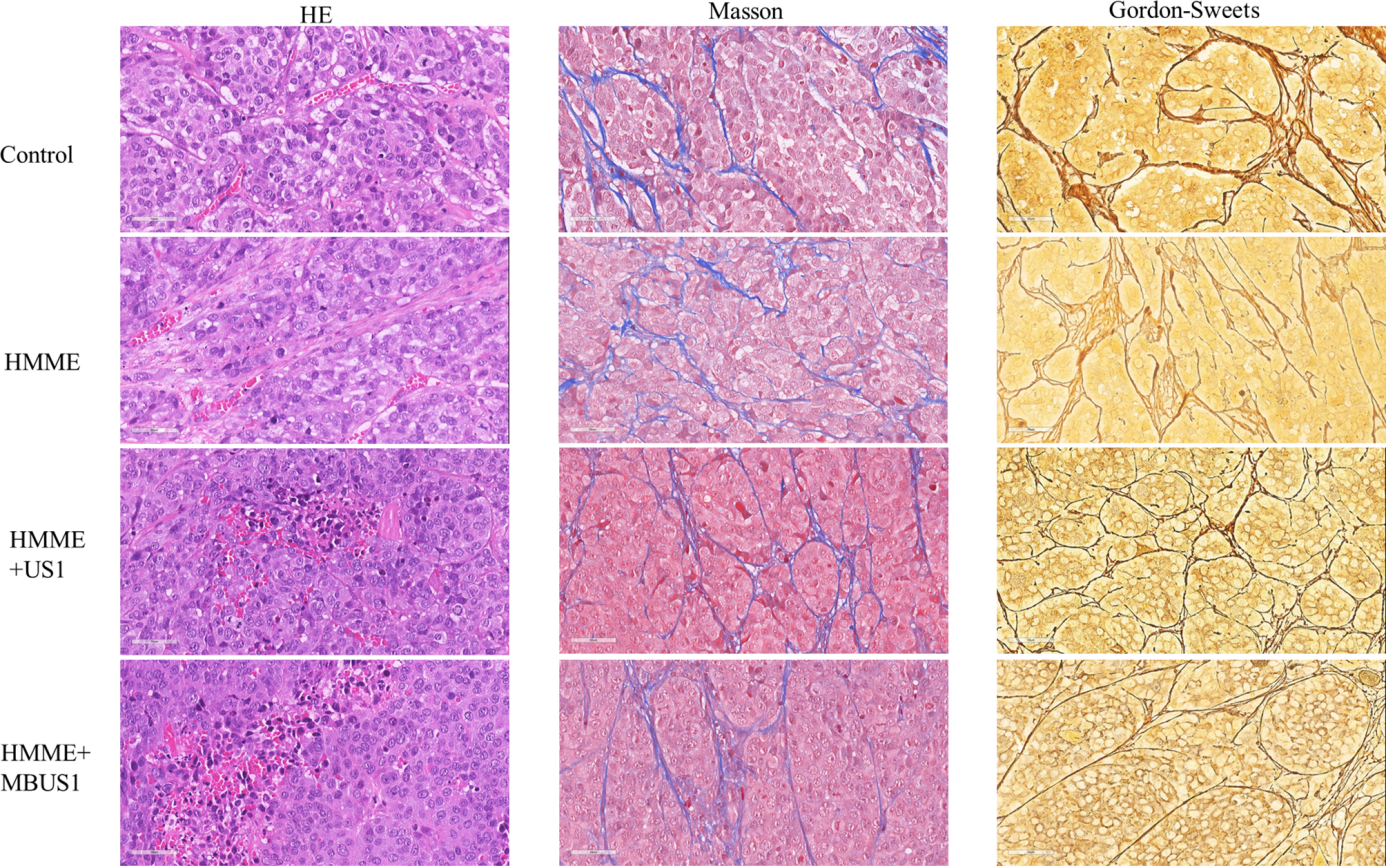
HE, Masson, and Gordon sweets stainings in each group after ultrasonic irradiation.

The HE staining results of tumor tissues showed that the pathological changes in the control and HMME groups were similar **(** **Figure 5**). The cells in the tumor tissue were disordered and distributed in a strip-like manner. In each proliferative stage, tumor cells were dense and structurally complete. Meanwhile, passing blood vessels were detected, branching or cystic, with a clear structure, complete and continuous pipe walls, without clear damage, with red blood cells in the lumen and around the blood vessels. No clear red blood cell escape was found. In the HMME + MBUS1 group, tumor cells were disorderly distributed with few nuclear pyknosis and tumor microvessels distributed. Among them, a small amount of red blood cell leakage was scattered around blood vessels. In the HMME + MBUS1 group, flake necrotic foci were also seen in the sparse tumor tissues, and clear nuclear pyknosis or fragmentation accompanied by a large number of nuclear fragments were detected. Microvessels were congested and dilated, the blood vessel wall was incomplete, and a large number of red blood cells escaped around the blood vessel. The Masson staining showed that the collagen fibers were blue, the blood cells and cytoplasm red, and the nucleus blue-purple **(** **Figure 5** **)**. Moreover, no significant correlation between ΔIFP and the content of collagen fibers in tumor tissues was detected in each group. The Gordon sweets reticular fiber staining showed black hairy reticular fibers in all pathological sections. The content of reticular fibers in tumor sections was similar between groups and did not correlate with the ΔIFP.

### Therapeutic Effect of SDT

**Figure 6:**
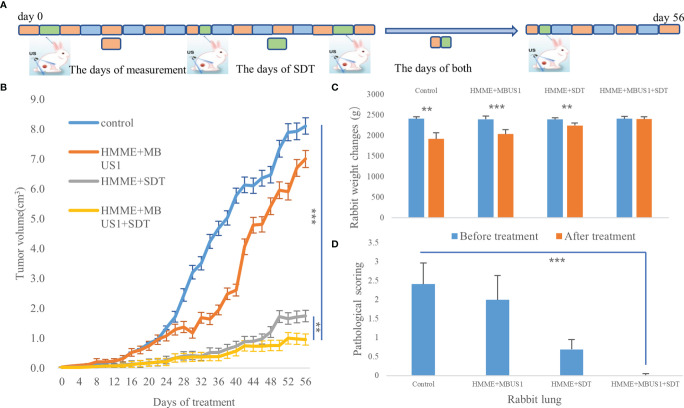
**(A)** The days of measurement, SDT, and both of them. **(B)** Tumor volume changes with time after treatments. **(C)** Changes of rabbits body weight before and after SDT (**p < 0.01,***p < 0.001). **(D)** Pathological scorings of rabbits lung metastasis (***p < 0.001).

**Figure 7:**
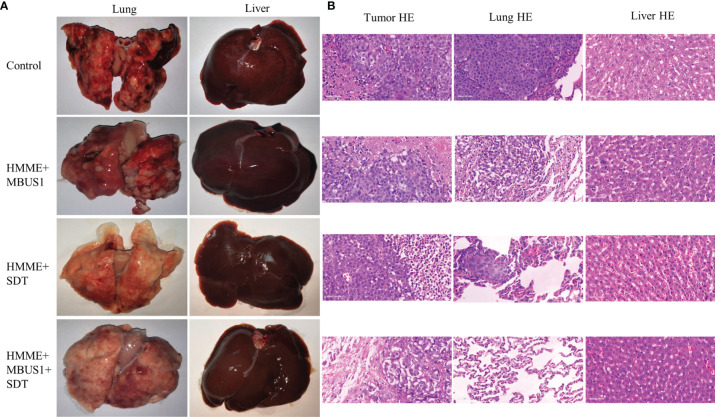
**(A)** Liver and lung metastasis of tumor-bearing rabbits in each group after treatment was observed by the naked eye. **(B)** Tumor tissue, lung, and liver of each group were observed by HE staining.

The tumor growth curve results showed a significant difference in tumor volume between groups two days after the first treatment (*F* = 7.432*, p* = 0.002). The tumor volume of the blank group presented the fastest increase followed by the HMME + MBUS1 group. The slowest tumor growth was observed in the HMME + MBUS1 + SDT group **(** **Figure 6B** **)**. Moreover, the weight change of rabbits in **Figure 6C** testified that the rabbits weight had no noticeable change over the course of the experiment. Pathological sections were scored In order to quantitatively evaluate lung and liver metastasis, 10 different high-power fields were randomly observed in each section, and the score was based on the positive cell rate. The scoring criteria were as follows: 0 points, no metastatic cells were observed. Score 1, 2, 3 and 4 were positive cell rates of 1%-25%, 26%-50%, 50%-75% and 76%-100% respectively **(** **Figure 6D** **)**. The anatomical specimens showed that the blank group lungs were covered with miliary metastases of different sizes, Multiple white metastases were also seen in the lungs of the HMME + MBUS1 group. In these groups, large metastases were also present in lung tissues (**Figure 7A**) with the pathological scorings of 2.41 and 2.00. No clear metastasis was detected for the HMME + SDT group using the naked eye, but the staining showed occasional punctate metastases in the lung tissue, the pathological scorings of this group is 0.69. What’s more, We only see one field with a pathological scoring of 1 point among 15 lung tissue sections in the group of HMME + MBUS1 + SDT group **(** **Figure 7B** **)**. Finally, the liver tissues of tumor-bearing rabbits in each group did not present metastasis with pathological scoring of 0.

## 4 Discussion

Compared with photodynamic therapy, SDT has deeper tissue penetration, higher precision, fewer side effects, and good patient compliance. Therefore, it has a good application prospect for deep solid tumors such as liver cancer, glioma, etc. (18). Moreover, the treatment can be repeated, being especially suitable for elderly and weak cancer patients who cannot undergo surgery, radiotherapy, or chemotherapy (19). Our study was aiming to enhance SDT by using microbubbles combined with ultrasonic cavitation to increase the penetration of sonosensitizers into tumors.

The accumulated concentration of sonosensitizers in tumors limits SDT’s clinical application. HMME is the most commonly used photosensitizer and sonosensitizers in photodynamic and sonodynamic therapies (20). For example, Liang et al. (21) reported the HMME-SDT synergistic effect with the anticancer agent DOX. The combined application of HMME-SDT and DOX significantly inhibited the proliferation of human cholangiocarcinoma QBC939 cells *in vitro*. Moreover, HMME has advantages such as single composition, stable performance, high tumor selectivity, and low phototoxicity to normal tissues. but a high tumor IFP hinders the accumulation of sonosensitizers in tumor sites (22). Due to tumor vascular heterogeneity, the IFP in tumor tissues can be increased by normal, dense stroma and abnormal fibrosis, abnormal function of collagen fibers and reticular fibers, and abnormal lymphatic vessels, which hinder the material from entering the tumor stroma from capillaries **(** **Figure 1**).Tumor IFP increases (23) can be caused by abnormal tumor blood vessels and dense interstitial matrix and abnormal fibrosis, increased hyaluronic acid in the interstitial matrix, and abnormal lymphatic vessels. In the present study, before treatments, the pressure in the central part of the tumor was the highest, showing a downward trend from the center to the periphery. However, the tumor IFP was still positive and the pressure in the surrounding muscle tissue was negative.

The main methods to reduce tumor IFP are to “normalize” blood vessels. Besides, reducing the content of hyaluronic acid in the interstitial matrix and improving lymphatic function can increase the efficacy of antitumor drugs. In the current experiment, the combination of microbubbles with low-frequency unfocused ultrasound irradiation could reduce the tumor’s IFP. We believe that this was possible due to: first, the cavitation effect **(** **Figure 1**). Microbubbles generate cavitation under ultrasound irradiation, resulting in a shock wave and microjets, resulting in tumor vascular damage, rupture, and micro thrombosis, thereby destroying the blood perfusion of the tumors and causing necrosis and apoptosis of tumor cells. At the same time, the vibration and explosion of microbubbles in blood vessels can destroy the tumor microvascular structure, damage endothelial cells, and even lyse cells. Second, As confirmed by our pathological images, the destruction of tumor blood vessels reduces the microvascular area in the tumor, consistent with our ultrasound imaging results before and after cavitation. After cavitation, the PI value of all tumors’ central parts decreased in the experimental group **(** **Figure 3**), as the tumor blood flow and macromolecular substances escaped from blood vessels, finally reducing the tumor IFP. Third, cavitation caused the destruction of tumor vascular structure and induced apoptosis and necrosis by disrupting the integrity of the endothelial cytoskeleton. Besides, endothelial cell necrosis can indirectly reduce tumor IFP. Previous studies indicated that the tumor IFP is positively correlated with the content of collagen and reticular fibers in tumor tissues. Here, the tumor IFP changed after the combination of low-frequency ultrasound with microbubbles irradiation, but the structure and content of collagen and reticular fibers in each group did not significantly change.

Tumor cells produce mechanical forces in the process of excessive growth and reproduction, which compress tumor blood vessels and lymphatic vessels, reduce microcirculation blood perfusion, and increase tumor IFP. In this project, it was found that ultrasonic cavitation of microbubbles could increase the permeability of tumor microvessels to sonosensitizers, thus increasing the accumulation of sonosensitizers at the tumor site **(** **Figure 4** **).** HE staining showed obvious direct damage to vascular endothelium at 2.5MPa sound pressure, incomplete structure of vascular wall, and overflow of red blood cells from the rupture into the surrounding blood vessels **(** **Figure 5** **)**. Partial tumor vascular structure is incomplete, and the tumor cells around the blood vessels appear scattered focal necrosis. Confocal laser microscopy **(** **Figure 4** **).** showed that the content of HMME in the cavitation group increased in the edges of the tumor with relatively abundant blood vessels, which may be because HMME entered the extravascular space through the enlarged vascular space, and it was difficult for HMME to flow in the dense extravascular matrix and thus accumulated here. Collagen fiber and reticular fiber is an important component of the extracellular matrix of tumor, how much of its content is also an important factor affecting the tumor IFP, pathological section shows each tumor in the structure and content of collagen fiber and reticular fibers and no obvious change **(** **Figure 5**), possible reason is that blood vessels has played a more important role in regulating tumor IFP, This also indicates that microbubbles combined with ultrasonic cavitation can effectively change tumor IFP without causing changes in skeletal structure such as collagen fibers and reticular fibers in tumor extracellular matrix.

One hour after HMME intravenous injection at the rabbit ear edge, the HMME content in tumor tissues was at a high level. Then, SonoVue microbubbles were intravenously injected and reached tumor tissues. Under ultrasound stimulation, the microbubbles produce a cavitation effect, which can temporarily form acoustic holes in the blood vessel wall or cell membrane. The diameter of these holes ranges from a few nanometers to 150 nm. They can enhance the permeability of blood vessels and cell membranes, then promote drug penetration and cellular uptake in the treatment area. Combining ultrasound with microbubbles to enhance chemotherapeutic drugs is also known as sonochemotherapy (24). Ultrasound-enhanced chemotherapy drug release only occurs in the ultrasound irradiation area, and the therapeutic drug concentration increases specifically in the focus area, resulting in significant therapeutic response, which can also reduce the side effects of drugs in other parts. Sonochemotherapy has been used in the clinical treatment of various solid tumors, such as prostate cancer (25), melanoma (26), and pancreatic cancer (27). In this study, there was no obvious change in tumor blood perfusion at the 1/4-1/8 tumor edge, while IFP decreased significantly. Qualitative and quantitative analysis showed that the accumulation of HMME in this region increased. Then, we performed the second step ultrasound SDT treatment at this time and achieved a good therapeutic effect. The exact and definitive mechanism of SDT remains unresolved. Possible theories include generation of ROS, ultrasonic cavitation effect and thermal destruction (28).

At present, 0.15-2.0 MHz ultrasound is generally used during SDT. The normal irradiation amount is 2-3 W/cm^2^ for 60 s-30 min (29). High-frequency ultrasound with high ultrasound intensity can produce a thermal effect and directly kill cells. At the same time, this might lead to increased reactive oxygen species (ROS) in the normal tissue around the tumor and cause irreversible damages (30). Low-frequency ultrasound has deeper penetration, which can temporarily enhance the cell membrane permeability, protect the surrounding normal tissues. Also, tumor cells in the cell proliferation stage are more sensitive to reactive oxygen species. Additionally, compared with high-frequency ultrasound, low-frequency ultrasound produces a larger cavitation bubble radius and greater spatial-temporal intensity of bubble rupture. In the current study, the SDT was conducted with 3 W/cm^2^, single treatment for 15 min, for 8 times. Results showed a clear tumor inhibition effect in the treatment group **(** **Figure 6** **)**. Ninomiya et al. found that under the same conditions when TiO_2_ was irradiated with two ultrasonic waves with different energies and frequencies (0.5 MHz and 800 MW/cm^2^, and 1 MHz and 0.4 W/cm^2^), more active hydroxide was produced, better than one ultrasonic wave (31). Therefore, the combination of ultrasounds with different energies and frequencies can lead to better therapeutic effects than single ultrasound. Two ultrasound frequencies were also used in the present experiment after the injection of an acoustic sensitizer. On the other hand, it is not clear whether ROS will be produced in the first ultrasound step. However, based on the tumor growth curve **(** **Figure 6** **)**, the growth rate of the HMME + MBUS1 group was only the second, after the blank control group, which was significantly faster than the HMME + MBUS1 + SDT group. Therefore, we speculated that the main reason for the tumor tissue growth inhibition was the second SDT step. The first step of the HMME + MBUS1 mainly reduced tumor IFP and increased the accumulation of the acoustic sensitizer at the tumor site. SDT utilizes the interaction between ultrasound and non-toxic acoustic sensitizers, which selectively accumulate in the target tissue, eradicating solid tumors in a non-invasive and highly selective way.

However, SDT still has some problems requiring further studies:(I) The specific mechanisms of SDT for cancer treatment are not completely clear; (II) New sonosensitizers with less phototoxicity and higher therapeutic effects need to be explored;(III) The ultrasound frequency, intensity, and irradiation time corresponding to specific tumors still need to be studied in more detail;(IV) Long-term toxicity studies of existing sonosensitizers need to be performed in the future. Finally, due to its good biomedical performance, SDT has attracted increasing attention from many cutting-edge interdisciplinary areas related to cancer. We believe that SDT will shortly have a great impact on the treatment of cancer patients.

## Data Availability Statement

The original contributions presented in the study are included in the article/supplementary material. Further inquiries can be directed to the corresponding author.

## Ethics Statement

The animal study was reviewed and approved by South China University of Technology.

## Author Contributions

FX and JL conceived and designed the experiments and wrote the paper; FX and YF performed the experiments and analyzed the data; LC and QC contributed reagents/materials/analysis tools. All authors provided their approval for publication.

## Funding

This work was supported by the National Natural Science Foundation of China (Grant No.: 82071935).

## Conflict of Interest

The authors declare that the research was conducted in the absence of any commercial or financial relationships that could be construed as a potential conflict of interest.

## Publisher’s Note

All claims expressed in this article are solely those of the authors and do not necessarily represent those of their affiliated organizations, or those of the publisher, the editors and the reviewers. Any product that may be evaluated in this article, or claim that may be made by its manufacturer, is not guaranteed or endorsed by the publisher.
